# A Case of Pulmonary Cryptococcosis Caused by Capsule-Deficient Cryptococcus neoformans

**DOI:** 10.7759/cureus.48196

**Published:** 2023-11-03

**Authors:** Brandol Wolfenbarger, Erin Britt

**Affiliations:** 1 Pathology, Alabama College of Osteopathic Medicine, Dothan, USA; 2 Internal Medicine, Cullman Internal Medicine, Cullman, USA

**Keywords:** histology and histopathology, pulmonary cryptococcosis, cryptococcus neoformans (c. neoformans), fungal lung infection, capsule deficient cryptococcus neoformans

## Abstract

Cryptococcosis, a fungal infection primarily caused by *Cryptococcus neoformans* (CN), is a significant concern for immunocompromised individuals. This paper presents a case of a 51-year-old immunocompromised male who initially presented with symptoms suggestive of community-acquired pneumonia but was later diagnosed with pulmonary cryptococcosis caused by capsule-deficient CN. The patient's exposure to construction dust, coupled with his immunocompromised state due to immunosuppressive treatment for psoriatic arthritis, likely contributed to his susceptibility. The unique presentation of the disease, due to the absence of the characteristic thick capsule, presented a diagnostic challenge. A brief review is provided looking at the mechanism, pathogenesis, and implications of capsule deficiency in CN. The case provides an example of one of the many presentations of cryptococcosis, especially in immunocompromised individuals, and highlights the diagnostic complexities of capsule-deficient CN strains.

## Introduction

Cryptococcosis is a fungal infection caused by the *Cryptococcus* genus having about 37 species, with *Cryptococcus neoformans* (CN) being the most commonly identified species. As an opportunistic pathogen, CN poses a significant threat to immunocompromised individuals. When present in the lungs, the disease’s non-specific clinical presentation and radiographic findings can easily be mistaken for other more common conditions. Pulmonary cryptococcosis caused by capsule-deficient CN is an infrequent diagnosis, made challenging by an abnormal morphology. This case report describes a 51-year-old immunocompromised male who presented with a clinical picture of sepsis secondary to bacterial pneumonia. Additional workup revealed that he had pulmonary cryptococcosis caused by capsule-deficient CN, illustrating the strengths and limitations of standard diagnostics.

## Case presentation

A 51-year-old Caucasian male presented to the emergency department (ED) with a two-month history of worsening dyspnea, productive cough, congestion, intermittent fevers, and myalgias unresponsive to outpatient treatments. The patient denied any headache, stiff neck, nausea, or neurological deficits. His medical history was significant for childhood seizures and psoriatic arthritis, the latter had been managed with upadacitinib and methotrexate for the past two years. He denied any tobacco, alcohol, or illicit substance use. Professionally, he worked in construction and experienced significant dust exposure over the preceding six months.

**Figure 1:**
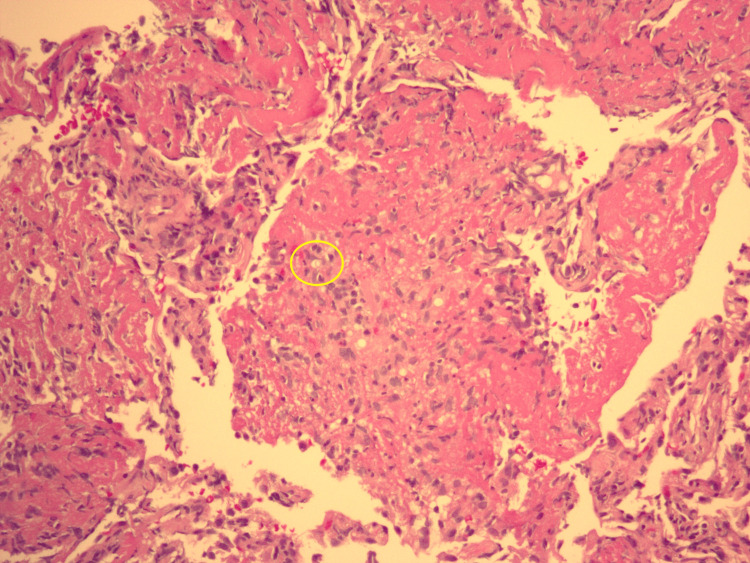
Section from a transbronchial lung biopsy viewed at 20x magnification, stained with hematoxylin and eosin, reveals a yeast-like organism (highlighted with a yellow circle)

**Figure 2:**
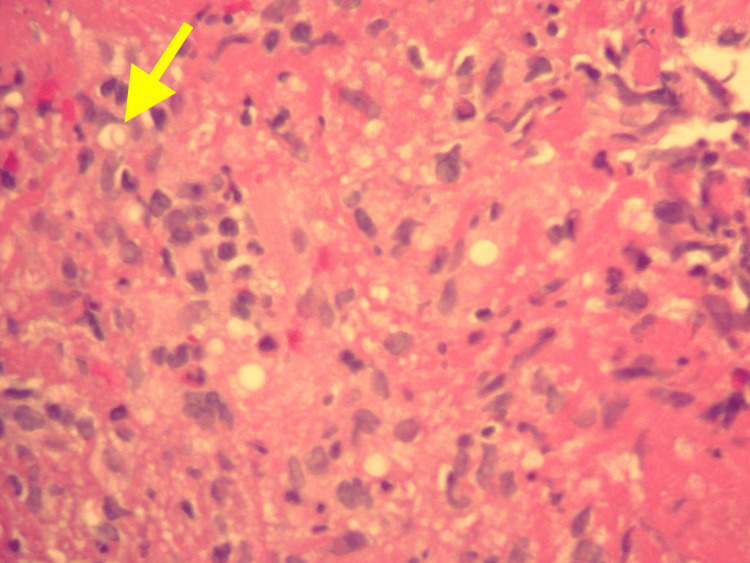
Section from a transbronchial lung biopsy viewed at 60x magnification, stained with hematoxylin and eosin, reveals a yeast-like organism (highlighted with a yellow arrow)

**Figure 3:**
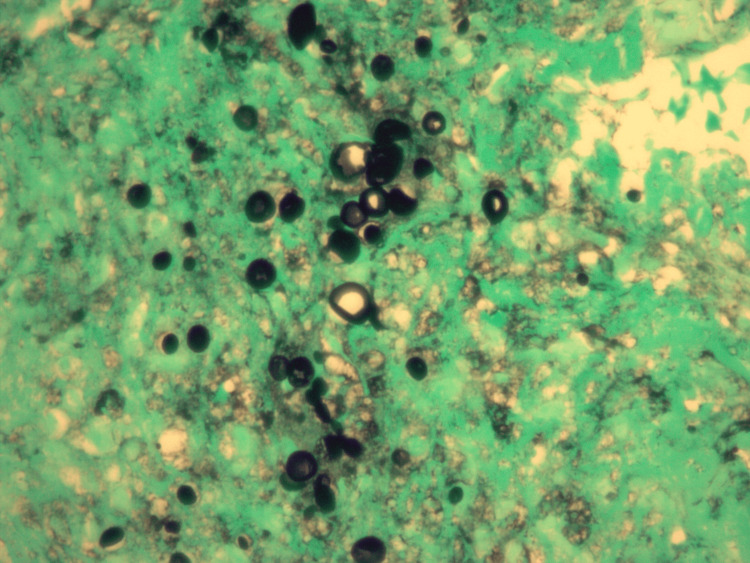
Section from a transbronchial lung biopsy viewed at 60x magnification, stained with Grocott's methenamine silver, reveals numerous yeast organisms

**Figure 4:**
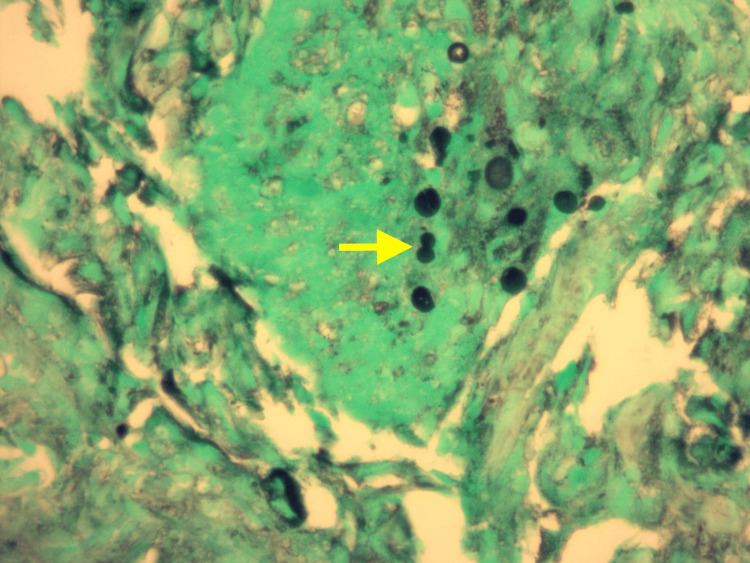
Section from a transbronchial lung biopsy viewed at 60x magnification, stained with Grocott's methenamine silver, reveals potential broad-based budding (highlighted with a yellow arrow)

A month prior, upon investigation by his primary care physician, a thoracic radiograph showed features suggestive of pneumonia, prompting empiric antibiotic treatment. Despite this, his symptoms worsened and he presented to the ED two weeks later. At that time, a viral panel tested positive for coronavirus HKU-1. Thoracic radiographs revealed patchy perihilar opacities in the right middle lung lobe, consistent with multifocal pneumonitis. After receiving IV ceftriaxone and azithromycin, he was prescribed a 10-day course of oral prednisone. However, his symptoms worsened, bringing him back to the ED two weeks later. Physical examination at that time revealed tachycardia and mild bilateral wheezing, without supplemental oxygen. The results of blood tests included an elevated white blood cell (WBC) count of 10.65 x 10^3/uL, an elevated lactic acid level of 2.3 mmol/L, and an elevated D-dimer of 0.63 mg/L fibrinogen equivalent units. Thoracic radiographs and computed tomography (CT) angiography of the chest indicated bilateral pneumonia, leading to hospital admission with the presumptive diagnosis of sepsis secondary to pneumonia. Initially, the treatment included IV ceftriaxone, IV azithromycin, and a combination of albuterol and ipratropium. Later, IV levofloxacin and IV steroids were added to the regimen. Despite marginal symptomatic improvement for some days, an overall decline in his condition was evident with worsening radiographic findings and a rise in daily WBC count, peaking at 17.75 x 10^3/uL. In addition, he progressed from room air to requiring four liters of oxygen per minute by nasal cannula. Flexible bronchoscopy was performed which revealed extensive purulent discharge. Slides stained with hematoxylin and eosin (H&E) from sections of formalin-fixed paraffin-embedded tissue blocks of a transbronchial biopsy of the right upper lung revealed benign bronchial epithelium and alveolar tissue with a few organisms identified in a background of eosinophilic proteinaceous material. These findings are shown at low magnification (Figure 1) and higher magnification (Figure 2). Grocott's methenamine silver (GMS) staining highlighted individual and clustered yeast forms of varying sizes (Figure 3), with possible broad-based budding observed in only one focus (Figure 4). Mucicarmine staining for capsular material was negative. While *Blastomyces dermatitidis* was initially suspected because of possible broad-based budding and lack of a thick capsule, subsequent testing was pursued. *Blastomyces *antigen testing yielded negative results in the following days. Cryptococcal antigen (CrAG) lateral flow immunoassay titers were found to be greater than or equal to 1:2560 and bronchial washings culture grew *C. neoformans*, confirming the diagnosis as cryptococcosis. Following the introduction of IV fluconazole, the patient’s clinical status markedly improved. WBC counts returned to normal range over the remainder of his hospitalization. After a 22-day hospital stay, he no longer required oxygen supplementation and was discharged with a prescription for 200 mg of oral fluconazole twice daily for 6-12 months, under the supervision of his primary care physician. After 42 days of incubation, cultures for *Mycobacterium* showed no growth. Five months post-discharge, a follow-up thoracic radiograph displayed stable atelectasis in the right middle lobe and discernible scarring in the left lung base.

## Discussion

We present a case of pulmonary cryptococcosis caused by capsule-deficient *C. neoformans* in an immunocompromised host. The abnormal morphology complicated histologic staining and identification, but a positive tissue culture and serologic studies confirmed the diagnosis. Following an extended course of oral fluconazole, the patient showed no signs of relapse at a five-month post-discharge follow-up. In many ways, capsule-deficient CN is clinically very similar to its capsule-intact counterpart. However, the differences that do exist may influence the patient population, progression of disease, and success of standard diagnostics.

Background

The genus *Cryptococcus* consists of over 30 classified species, all of which are characterized by their encapsulated yeast form [1]. Taxonomic classification of the pathogenic cryptococcal species has been a subject of debate. However, recent phylogenetic studies have suggested rearranging the classic divisions. In this new classification, serotype A and AD fall under *C. neoformans*, serotype D is categorized as *Cryptococcus deneoformans*, and the *Cryptococcus gattii *group is divided into five distinct species [1,2]. Historically, from a clinical perspective, the primary focus has been on *C. neoformans* and *C. gattii,* as they are the primary pathogenic species in humans [1,3]. CN is a globally distributed, facultative intracellular, saprophyte predominantly found in soil enriched with avian excreta, especially that of pigeons, likely due to the high nitrogen content that supports its growth [2,4,5]. It can also be found in areas with decaying organic material, including wood, milk, fruits, vegetables, and dust [4,6]. Morphologically, it is a thin-walled, non-mycelial, budding yeast characterized by its distinctive thick capsule, primarily composed of glucuronoxylomannan and galactoxylomannan [2,5].

Pathogenesis

In soil, CN exists in a yeast form, measuring about 3 micrometers, with minimal capsule present, if at all, due to the soil alkalinity and poor growth factors [6-9]. Studies suggest that human nasal passages effectively filter particles larger than 5 micrometers, with particles between 4-5 micrometers infrequently reaching the alveoli [8]. Encapsulated CN yeast cells, ranging from 5-20 micrometers [1], are too large to reach the lung alveoli, and thus infection is thought to occur through inhalation of non-encapsulated basidiospores, a smaller sexual spore form [10], or desiccated yeast cells [11], both capable of reaching the terminal bronchioles and alveoli. Bronchial secretions then facilitate the organism’s reproduction and capsule development [8]. In vitro studies in excised lung tissue have demonstrated that sufficient capsular development is accomplished within 5-10 hours to inhibit phagocytosis by 50% [8]. However, the host’s immune system often clears the infection before clinically significant disease progression occurs [7,9,10,12-14]. In many cases, phagocytized dormant yeast cells within phagolysosomes will establish a latent infection within the thoracic lymph nodes or a pulmonary granuloma that can remain asymptomatic for years until the host experiences some form of immunosuppression allowing it to reactivate [1]. Once formed, the capsule protects the yeast from host immune response by depressing antibody formation, resisting phagocytosis, and inhibiting migration inhibition factor formation [13]. Host immune responses typically exhibit mild or no tissue inflammation due to the capsule’s protective effect [10]. When the organism lacked a capsule, phagocytosis was approximately three times as effective, supporting the role of the capsule in preventing phagocytosis [14]. Capsule deficiency can arise either due to an innate inability that presents as a dry and wrinkled appearance on mycologic media or from chronic lesions where the host’s immune defenses strip away the capsule, as evidenced by capsular debris in surrounding phagocytic cells [3]. The inability to form a capsule inside a host, even under appropriate conditions, may stem from a mutation or a deficiency in the required metabolic pathway [7,15]. Experimental studies by Farmer and Komorowski demonstrated that capsule-deficient organisms isolated from humans did not exhibit in vivo capsule development, indicating a potential metabolic pathway issue [7]. The loss of the capsular component in these deficient strains exposes underlying protein antigens, leading to increased host inflammatory response. This results in early suppuration, phagocytosis, and granuloma formation, which can exacerbate clinical disease manifestation [9-11,13]. In experimental mouse models, intracerebrally injected capsule-deficient CN has been shown to evoke a robust immune response characterized by histiocytic and fibroblastic reactions that wall off the infection, resulting in a chronic inflammatory response [7,12]. Despite capsule deficiency, in theory, leading to more severe disease, both encapsulated and non-encapsulated variants have been found to have comparable outcomes with similar treatment modalities [16]. Other virulence factors, including melanin production, growth at 37°C, mating type, efficient iron utilization, and secretion of enzymes like proteases, laccase, and urease, also contribute to the pathogenicity of CN [16].

Clinical presentation

The presentation and development of symptoms from a CN infection depends on the size of the inoculum, site of infection, variance of the strain, and the host’s immune response [10]. CN infections are overall more common in men [6]. The central nervous system (CNS) is the most frequently affected site, followed by the lungs [10]. The majority of infected patients have some form of underlying cell-mediated immune deficiency, which could result from various medical conditions, malignancies, or medications such as steroids and other immunosuppressants, as evidenced by this case [6,10,12]. While a large enough inoculum could infect a healthy host, adequate T-cell function should result in rapid clearing of the infection given the lack of the capsule [6,11]. This distinction can aid in diagnosis as histoplasmosis and blastomycosis infections are not as strongly correlated with immunocompromised hosts [12]. The patient in this case was immunocompromised secondary to the combination of upadacitinib and methotrexate for the treatment of his psoriatic arthritis. Immunocompromised patients are more likely to be symptomatic and to have dissemination to the CNS, skin, and bones [5]. Rarely, Pancoast’s syndrome and chest wall involvement have been reported in cases of pulmonary CN infections [10]. The presenting symptoms of pulmonary cryptococcosis are non-specific: 54% of patients experience cough, 46% report chest pain, and 32% have sputum production [6]. Boyars et al. found that 32% of patients are asymptomatic, and fever is present in only 26% of infected individuals [6]. Other reported symptoms include fever, dyspnea, night sweats, malaise, weight loss, hemoptysis, and pleuritic chest pain [6,10,11,15].

Diagnosis

Diagnosing capsule-deficient CN-related pulmonary cryptococcosis requires an understanding of the approach to standard encapsulated strains and how capsule deficiency can make routine testing complicated depending on the degree of deficiency. There are limited conclusions available in research contrasting capsule-deficient CN and capsule-intact CN. As with capsule-intact CN, microscopic examination of sputum, bronchial washings, biopsy, or fine needle aspirations might reveal organisms [11]. However, abnormal morphology and low organism count due to increased susceptibility to host immune response complicate histological identification and organism viability for culturing [7,10]. Histologically, *Cryptococcus *appears as globular, usually encapsulated, yeast cells with or without budding, ranging in size from 5 to 20 micrometers in diameter depending on capsule thickness [1]. Stains like Periodic acid Schiff, GMS, and calcofluor white can be helpful for highlighting *Cryptococcus *given its poor staining with H&E [2]. Traditionally, India ink has been used to identify capsule-intact CN, but it has a low sensitivity compared to CrAG tests and it relies on the presence of the capsule to distinguish CN [2]. While capsule-intact CN is highlighted red with mucicarmine, this stain is less effective as the degree of capsule deficiency increases [9]. With electron microscopy and the use of immunofluorescence, it appears to be rare to encounter an infection in which you will not find at least some cells possessing a detectable capsule; however, these modalities are costly and not always readily available [3,12,17]. In an attempt to standardize the criteria for capsule deficiency, Torres et al., in a small retrospective study, defined capsule deficiency as isolates possessing a capsule thickness less than or equal to 1 micrometer as identified by histopathologic analysis based on standard staining procedures [16]. Morphologically, capsule-deficient CN may resemble immature (non-endosporulating) *Coccidioides *[3], *Blastomyces*, or *Histoplasma *[10]. Similarly, they do not stain with mucicarmine [10]. *Coccidioides *can be more easily distinguished as it will likely include sporangia along with sporangiospores and spherules at any stage of disease [3]. *Blastomyces *and *Histoplasma *offer the most similar appearance, but Fontana-Masson stain (FMS) can effectively differentiate CN from these organisms regardless of capsule presence [3,9,10] Originally developed by Fontana in 1912 and later modified by Masson in 1928, it detects melanin and other silver reducing substances. CN possesses the enzyme phenol oxidase which, in the presence of dihydroxy or polyhydroxy phenols in the substratum, produces a melanin-like pigment that accumulates in the cell wall and is highlighted by FMS [9,10]. *Sporothrix*, another common pathogenic fungus, has some similar staining characteristics; however, it has a distinct cigar shape and stains a light brown when exposed to FMS in contrast to the dark brown of CN [3,10]. Pulmonary cryptococcosis can manifest as a variety of imaging findings, ranging from a single ill-defined mass to multiple round opacities, with varying degrees of infiltrates or effusions [11]. In immunocompetent hosts the most common radiographic finding is well-defined, non-calcified pulmonary nodule(s) that may be mistaken for malignancy [10]. CT scans frequently reveal nodules, predominantly in the upper and middle lung zones, alongside focal consolidations [5]. However, other findings have been reported including diffuse reticulonodular opacities, pleural effusion, hilar and mediastinal lymphadenopathy, and cavitation within pulmonary nodules (10-15%) [5,10]. Immunocompromised hosts exhibit similar findings but can progress to more severe cases showing a miliary pattern or a pattern suggestive of adult respiratory distress syndrome [9]. AIDS patients specifically will often have mediastinal and hilar lymph node enlargement rather than pulmonary nodules [5]. Imaging findings specific to capsule-deficient CN have not been widely studied and no consensus is currently available. Nodule formation is, in theory, dependent on the presence of a capsule for its anti-phagocytic property [10].

CrAG tests, designed to detect shed cryptococcal polysaccharide capsular antigen, have shown varied results in studies. Some capsule-deficient CN strains have tested positive despite the absence of a visible capsule, while others have yielded the expected negative results [12,16]. For CN infections, latex agglutination techniques have been the most commonly used for this serologic test until recently, and in the case of serum and CSF, have a sensitivity and specificity of 93% to 100% and 93% to 98%, respectively [1]. False negatives with latex agglutination may occur due to the postzone effect, low fungal load, or lack of capsule [2]. CrAG latex agglutination is falling out of favor due to refrigeration requirements, 45-minute turnaround, and lower sensitivity for non-HIV related or *C. gattii *patients [2]. The CrAG lateral flow immunoassay offers a rapid, low-cost, highly sensitive, and specific (greater than 98% for serum and CSF) alternative that has a faster turnaround (15 minutes), minimal infrastructure requirements, wider capture of *C. gattii* polysaccharides and is stable at room temperature [1,2]. Elevated titers in cases of diagnosed capsule-deficient CN may be due to an inconsistent or minuscule capsule not easily visible on light microscopy or organisms that had their capsules stripped away by host immune response and now appear non-encapsulated [3]. If present, titers greater than or equal to 1:4 are strongly suggestive of disease [10]. Higher than 1:64 has been associated with extrapulmonary dissemination in non-AIDS patients [10]. High titers and neurological symptoms should prompt a workup for CN meningitis, particularly in the non-AIDS population if not already done [10,11]. CN-infected patients with AIDS typically exhibit strongly positive antigen tests regardless of the infection site [11]. In the immunocompromised host, recommendations have been to assume dissemination regardless of antigen and proceed to a lumbar puncture, and if negative then the disease is typically self-limiting [11]. Serial quantitative estimation of capsular polysaccharide in serum may serve as a prognostic factor, though its relevance may be diminished by variance in host immune response or capsule deficiency [13]. Biopsy of a lung mass in patients with negative extrapulmonary and serologic workup may be warranted [6]. Regardless of the capsule, CN can be cultured on various agar media with Sabouraud’s dextrose agar showing a characteristic white and creamy mucoid growth [2]. Other options include inhibitory mold agar, brain heart infusion agar, bird seed agar, and blood agar plates with growth appearance differing by culture media [2]. Culture growth can range from a few days to 4-6 weeks, with capsule production typically beginning after 10 days [2,6,11]. Laboratory submissions should specify that CN is a consideration due to its sensitivity to cycloheximide [2]. Due to the frequent isolation of CN in sputum and bronchial secretions without evidence of invasive disease, the value of cultures diminishes in isolated pulmonary infection scenarios [12].

Treatment

While treatment does not seem to vary based on the presence of a capsule, specific research on the treatment of capsule-deficient CN is lacking. The standard anti-fungal regimen for CN infections typically involves amphotericin B, flucytosine, or oral azoles [2]. The Infectious Disease Society of America recommends that symptomatic patients with a serum cryptococcal antigen titer exceeding 1:8 and positive lung cultures initiate therapy with oral azole drugs [10]. Those with mild to moderate symptoms are typically prescribed fluconazole, at dosages ranging from 200 to 400 mg/day for 6-12 months [10]. If fluconazole is not well tolerated, itraconazole can be used at comparable dosages [10]. For cases that are severe or resistant to azoles, the recommended treatment regimen begins with a combination of amphotericin B and flucytosine for at least two weeks. This is followed by an eight-week consolidation therapy using only flucytosine. Finally, a maintenance therapy of at least one year is recommended, with the dosage decreasing in each subsequent phase of treatment [2,10]. High-dose fluconazole is an appropriate alternative for flucytosine, and both allow a lower dosage of amphotericin B to be used. Clinical trials have shown that a single high-dose infusion with liposomal amphotericin B followed by fluconazole and flucytosine is as effective as a longer course of amphotericin B and has the benefit of a less severe side effect profile [2]. Adjustments for more severe diseases mostly rely on increasing the dosing or duration of amphotericin B [18].

## Conclusions

In conclusion, this report presents an uncommon case of cryptococcosis caused by capsule-deficient CN, manifesting as persistent pneumonia symptoms in an immunocompromised individual. This paper highlights the diagnostic challenges of CN, especially when it lacks the characteristic capsule and the role of histologic and serologic techniques. Capsule deficiency renders visual identification difficult, and depending on the mechanism, may affect culture viability. In addition, there is no clear consensus on the sensitivity of CrAG testing in these unique cases. With few studies on capsule-deficient CN, we hope our contribution helps in better understanding and diagnosing such cases in the future.
